# Elevated Fat Intake Increases Body Weight and the Risk of Overweight and Obesity among Chinese Adults: 1991–2015 Trends

**DOI:** 10.3390/nu12113272

**Published:** 2020-10-26

**Authors:** Liang Wang, Huijun Wang, Bing Zhang, Barry M. Popkin, Shufa Du

**Affiliations:** 1 Department of Public Health, Robbins College of Health and Human Sciences, Baylor University, Waco, TX 76798, USA; liang_wang1@baylor.edu; 2 Chinese Center for Disease Control and Prevention, National Institute for Nutrition and Health, Beijing 100050, China; wanghj@ninh.chinacdc.cn (H.W.); zhangbing@chinacdc.cn (B.Z.); 3 Department of Nutrition and Carolina Population Center, University of North Carolina at Chapel Hill, Chapel Hill, NC 27599, USA; popkin@unc.edu

**Keywords:** fat intake, body weight, overweight and obesity, longitudinal analysis, China

## Abstract

Studies on fat intake and obesity have been inconclusive. This study examined the associations between dietary fat intake and body weight and the risk of overweight and obesity in China. We used data from 23,859 adults aged 20–60 years who participated in the China Health and Nutrition Survey, an ongoing open-cohort study, from 1991 to 2015. We collected detailed dietary data by conducting three 24-h dietary recalls and weighing foods and condiments in household inventories. We examined the associations between fat intake and body weight, body mass index (BMI), and the risk of overweight and obesity with random-effects linear or logistic regression models for panel data. The Chinese population’s fat intake, percentage of energy intake from fat, and prevalence of high-fat diets (energy intake from fat > 30%) increased from 68.5 g per day (g/d), 23.1%, and 22.4%, respectively, in 1991 to 79.3 g/d, 35.6%, and 67.2%, respectively, in 2015. The prevalence of overweight and obesity increased from 12.3% to 37.3% during the same period. Fat intake, percentage of energy intake from fat, and a high-fat diet were positively associated with body weight, BMI, and the risk of overweight and obesity in both sexes (*p* < 0.001) after adjustment for nonfat energy intake, physical activity, and socioeconomic status. Increased fat intake and high-fat diets were associated with increased body weight, BMI, and risk of overweight and obesity. These findings could have a significant impact on Chinese policies and interventions to control overweight and obesity.

## 1. Introduction

Obesity has nearly tripled worldwide since 1975, and in 2016 over 1.9 billion adults were overweight or obese [1]. While the increase in overweight and obesity is global [2], the speed of change has been faster in many low- and middle-income countries. China has seen this rapid increase not only in overweight and obesity but also in major noncommunicable diseases [3,4,5]. Many lifestyle risk factors, such as an unhealthy diet, a major reduction in physical activity, and increased sedentary behaviors, may have contributed to the rapid rise in health issues in China [6,7,8].

The findings of studies on the association between fat intake and obesity have been inconclusive. The World Health Organization (WHO) and the 2016 Dietary Guidelines for Chinese Residents recommend no more than 30% of total energy intake from fat [9,10], as some cohort studies [11,12,13,14,15] and one key randomized controlled trial [16] have shown that a high-fat diet is a major risk factor for excessive weight gain. The 2015–2020 Dietary Guidelines for Americans removed the limitations on dietary fat intake [17], because numerous studies have shown that both high-fat diets and low-fat diets can be linked to weight loss in Western countries. Other high-income countries and the Food and Agriculture Organization of the United Nations have kept their recommendations regarding total dietary fat intake at no more than 35% of total daily energy intake [18,19]. They have turned their focus to such dietary components as saturated fat and complex carbohydrates in their guidelines.

Following the dietary transition in Western countries, Chinese people are abandoning their traditional diet, which has been considered healthy and includes mostly rice and wheat and their products, limited vegetables and legumes, and few animal-sourced foods [20,21,22]. Like those in most other Asian and African countries, China’s dietary transition is represented by rapid increases in fat intake, especially fat from edible oils [23], additional animal-sourced foods [11,12], and more recently small increases in consumption of highly processed foods. Frying with edible oil has also increased rapidly [22,24,25]. As a result, total fat intake has increased significantly [24,26]. However, researchers have not examined the effects of this dietary fat change on overweight and obesity in China. 

To fill this gap in the literature, we examined the associations of the amount of fat intake, the percentage of energy intake from fat, and the consumption of a high-fat diet (defined as energy intake from fat > 30%) [27,28] with body weight, body mass index (BMI), and the risk of overweight and obesity in China. We used data from a nationwide long-term cohort study containing detailed dietary intake data from 1991 to 2015. To date, very limited studies have examined the association between a high-fat diet and weight status in the Chinese population.

## 2. Materials and Methods

### 2.1. Study Participants

**Figure 1:**
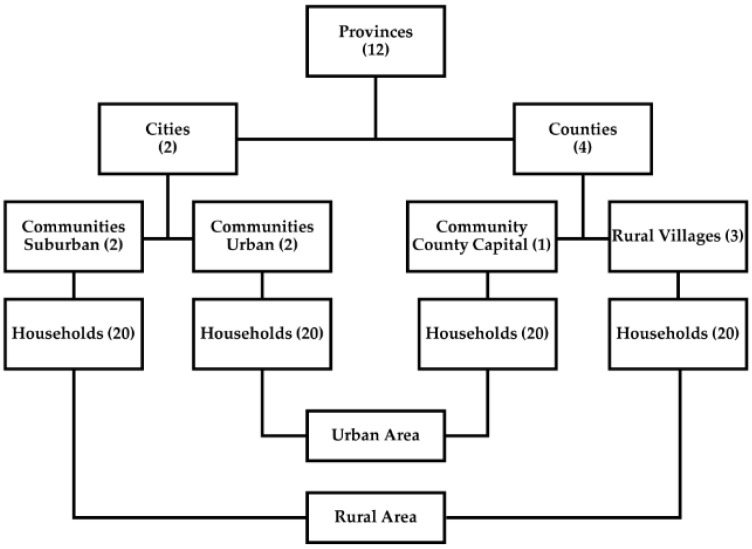
The sampling scheme used in the China Health and Nutrition Survey.

This study used data from the China Health and Nutrition Survey (CHNS), a 30-year ongoing open-cohort study initiated in 1989 and followed up through 2019. The overarching aim of this project is to examine how social and economic transformations in China have affected the health and nutritional status of its population. The survey used a multistage random-cluster sampling process to select samples from each of the nine provinces that vary in demography, geography, economic development, and access to public services [29,30] and represent all levels of socioeconomic development in China [31]. In each province, we stratified cities and counties by income levels and used a weighted-sampling scheme to randomly choose two cities (one larger high-income and one smaller low-income) and four counties (one low income, two middle income, and one high income). Then, we randomly selected two urban and two suburban neighborhoods in each city, one community in the capital of each county, and three villages in each county. Finally, we randomly drew 20 households in each selected area and interviewed all household members (Figure 1). When households were lost, we randomly selected additional households (and individuals in those households at any time) to replace them. We also recruited new households formed by the sampled individuals (e.g., divorce or marriage) from the baseline or subsequent surveys as replenishment samples. The design, sampling, and response rates have been reported in-depth elsewhere [29,30]. In 2011, we added the three largest cities in China (Beijing, Shanghai, and Chongqing) and used the same sampling schedule to draw samples from them. Overall response rates, based on those who participated in previous surveys and remained in the current survey, were above 80% at the individual level and above 90% at the household level.

This study followed adults aged 20 to 60 years (*n* = 23,859) who provided 65,029 observations to describe trends in fat intake and the prevalence of overweight and obesity. To assess the associations of fat intake with body weight and risk of overweight and obesity, we used a subsample of 14,562 adults who had provided diet and anthropometric data in the survey at least twice since 1991. They provided 55,732 observations, and each adult participated in the survey four times on average, varying from two to nine times. We did not use data from the CHNS 1989 or 2019, because the sample of dietary data was limited to young adults aged 20 to 45 years in 1989 and the 2019 data collection is still ongoing.

All subjects gave their informed consent for inclusion before they participated in the study. The study was conducted in accordance with the Declaration of Helsinki, and the protocol was approved by the Ethics Committee of the University of North Carolina at Chapel Hill (Project identification code 07-1963) and the National Institute for Nutrition and Health, Chinese Center for Disease Control and Prevention (Project identification code 201524).

### 2.2. Outcome Variables

Body weight, BMI, and overweight and obesity were our major outcome variables. Based on standard protocols recommended by the WHO [32], our trained physicians and nurses measured height to the nearest 0.1 cm without shoes using a portable stadiometer, and they measured body weight to the nearest 0.1 kg with lightweight clothing on a digital floor scale. We defined overweight as a BMI ≥ 25 kg per meter squared (kg/m^2^) and < 30 kg/m^2^ and obesity as a BMI ≥ 30 kg/m^2^ [32]. We combined overweight and obesity in the multivariable analyses, because the prevalence of obesity was very low in early survey years.

### 2.3. Exposure Variables

Fat intake, percentage of energy intake from fat, and a high-fat diet were the primary exposure variables, which we analyzed separately. We calculated these variables based on the diet data we collected and the compositions in the Chinese food composition table (FCT). We collected detailed dietary data (1) using three consecutive 24 h recalls for all foods, snacks, and beverages consumed at the individual level over the previous 24 h for each of the three days, whether at home or away from home; (2) weighing food items and condiments (e.g., edible oils and salt) added during food preparation and cooking at the household level over the same three-day period; and (3) taking food pictures, food samples, and a food diary, to help complete 24 h recalls. Interviewers went to each household daily and carefully recorded and measured all food items and condiments in the home inventory, whether purchased from markets or picked from home gardens, with Chinese balance scales (graduation 10 g) before 2004 and with digital diet and kitchen scales (graduation 1 g) thereafter at the start of the first 24 h recall and at the end of the last 24 h recall in each survey. Interviewers weighed and recorded all foods remaining after the last meal of the survey. When food was discarded and weighing was not possible, we used food pictures to help estimate the amount of food discarded. 

This combination of three-day 24 h recalls, household weighing, and food pictures and food diaries can improve the accuracy of recalls [33]. The three consecutive days during which interviewers collected dietary data were randomly spread from Monday to Sunday and were almost equally balanced across the seven days of the week for each county or city surveyed. All interviewers participated in at least one seven-day training session and passed a comprehensive test before collecting any data. Detailed dietary data collection and allocation have been described elsewhere [34,35]. 

### 2.4. Covariates

Interviewers recorded details of the time each household member spent per typical week in physical activities on weekdays and weekends, including occupational activity (e.g., light, moderate, and heavy), transportation activity (e.g., walking to and from work), domestic activity (e.g., buying food and cooking), and leisure sports activity (e.g., basketball and martial arts), and we converted the time to the metabolic equivalent of task (MET) based on the Compendium of Physical Activities [2,36,37] and dividied METs into three tertiles (low, middle, and high) in this study. Studies have demonstrated that lower METs for each component of occupation, transportation, household chores, and sports are predictive of weight gain [2,7,38,39,40,41]. We separated residency into two groups: urban areas, including communities in large cities and county capital cities, and rural areas, including communities in highly rural suburban areas and rural villages. We collected detailed data on incomes from all sources, including wages, farming, gardening, household businesses, and other income sources, inflated incomes to the 2015 Chinese yuan value, and divided inflation-adjusted per capita household income into tertiles, low, middle, and high-income levels, in all analyses. We divided education into two categories: below high school and high school or above. We defined smokers as those who ever smoked and alcohol drinkers as those who drank any alcoholic beverage at least once a month in the past year.

### 2.5. Statistical Methods

For continuous variables, we applied generalized linear regression models and tests to examine differences and trends. We tested categorical variables with chi-square tests. We used longitudinal random-effects (random intercept) linear regression models (xtreg, Stata command) or random-effects (random intercept) logistic regression models (xtlogit, Stata command) to analyze the associations of fat intake (controlling for total nonfat energy), percentage of energy intake from fat (controlling for total energy intake), and a high-fat diet (controlling for total energy intake) with body weight (controlling for height), BMI, and the risk of overweight and obesity. Additional covariates included age, sex, education, physical activity tertiles, income tertiles, residency, smoking status, drinking status, and survey year in all models. We specified individual identification code as the panel variable and survey year as the time variable to build models, estimated the relative risks (RRs) and 95% confidence intervals (CIs) from regression coefficients, and considered statistical significance as a two-sided *p* value < 0.05. We excluded participants who did not have body weight or fat intake data for all analyses and imputed missing income, education, and physical activity with the participant’s data available in the nearest survey year. We cleaned, managed, and analyzed all the data with SAS software (version 9.4, Cary, NC, USA) and Stata (version 14, College Station, TX, USA).

## 3. Results

We used 10 waves of CHNS data in all our analyses but presented only four waves of data in the descriptive Table 1 and Table 2 for brevity.

### 3.1. Sample Characteristics

The sample changed in composition over time (Table 1). In 1991, 17.4% of the participants had achieved a high school education, and in 2015 that percentage had increased to 41.1%. The proportion of urban residents significantly increased after 2011 (*p* trend < 0.001), when we recruited the three megacities. Occupation dramatically shifted away from labor-intensive activities, and physical activity largely declined, as the METs show (*p* trend < 0.001). The sex composition remained constant, but the age distribution shifted toward an older sample over time (*p* trend < 0.001).

### 3.2. Trends in Fat Intake and the Prevalence of Overweight and Obesity

On average, while total energy intake decreased by 26.1% (Table 2), from 2712.2 kilocalories per day (kcal/d) in 1991 to 2003.1 kcal/d in 2015, fat intake increased from 67.4 g per day (g/d) in 1991 to 78.3 g/d in 2015 (*p* trend < 0.001). As a result, energy intake from fat increased from 22.4% in 1991 to 35.3% in 2015. The percentage of people who consumed a high-fat diet more than tripled from 22.4% in 1991 to 67.2% in 2015 (*p* trend < 0.001). Men and women showed similar increases in energy intake from fat and high-fat diets, but men showed a higher fat intake and faster increase than women did each year (*p* trend < 0.001).

The major source of fat was edible oil, which accounted for 43.0% of total fat intake in 2015, down from 50.5% in 1991. Energy intake from edible oil increased from 11.1% in 1991 to 15.6% in 2015.

Average body weight increased 10.1 kg among men and 6.3 kg among women, while average height increased 2.6 cm among men and women during the 25-year period. Average BMI increased from 21.5 kg/m^2^ in 1991 to 24.4 kg/m^2^ in 2015 among men and from 22.0 kg/m^2^ in 1991 to 23.8 kg/m^2^ in 2015 among women. During the study period, the prevalence of overweight and obesity more than quadrupled among men, from 9.7% in 1991 to 41.9% in 2015, and more than doubled among women, from 14.7% in 1991 to 33.5% in 2015.

### 3.3. Associations of Fat Intake and a High-Fat Diet with Body Weight and the Risk of Overweight and Obesity

Table 3 and online supplemental Tables S1–S15 shows the associations of fat intake and the percentage of energy intake from fat with body weight and BMI and the association of a high-fat diet with the risk of overweight and obesity. Total fat intake, percentage of energy intake from fat, and a high-fat diet were positively associated with body weight, BMI, and the risk of overweight and obesity in both men and women. A 10 g/d increase in fat intake increased body weight by 0.033 kg per day (kg/d) (or 12.20 kg per year [kg/year]) among women and 0.023 kg/d (8.500 kg/year) among men (*p* < 0.001). A 10% increase per day in energy intake from fat increased body weight by 0.098 kg/d (or 35.60 kg/year) among women and 0.092 kg/d (33.50 kg/year) among men (*p* < 0.001). A high-fat diet increased the risk of overweight and obesity by 13.2% among overall participants (RR = 1.13, 95% CI: 1.04–1.23, *p* < 0.001), by 13.0% among women (RR = 1.13, 95% CI: 1.01–1.26, *p* < 0.001), and by 14.0% among men (RR = 1.14, 95% CI: 1.01–1.29, *p* < 0.001). The magnitudes of the coefficients for total fat intake and percentage of energy intake from fat were significantly different from the coefficients for total energy intake (*p* < 0.001). For example, in the body weight model for both sexes the difference was 0.032 (95% CI: 0.016–0.048, *p* < 0.001, results not presented).

## 4. Discussion

Using 10 waves of data from the CHNS, a nationwide cohort study, from 1991 to 2015, this study examined the trends in fat intake and their associations with the risk of overweight and obesity among Chinese adults. Unlike in the United States (U.S.) and other high-income countries, where fat intake has been constantly high in the past five decades [42], in China, fat intake and energy intake from fat, particularly energy intake from edible oil, and the percentage of people consuming a high-fat diet increased rapidly over the past 25 years, though total energy intake gradually decreased. In particular, fat consumption increased greatly among the lowest income tertile, as did overweight and obesity prevalence. Elsewhere we have studies that show the shifting burden toward the poor in China [43,44]. During the same period in China, the prevalence of overweight and obesity tripled. Our results show that increased fat intake significantly increases the risk of overweight and obesity in both Chinese men and women after adjustment for total nonfat energy intake, physical activity, and other potential confounding factors.

The prevalence of overweight and obesity increased rapidly in the past three decades in China, while total energy intake decreased. One of the reasons is that China experienced a major reduction in energy expenditure, which is measured in precise detail in the CHNS. Our results show that the METs in 2015 were only one-third of the levels in 1991. We have documented elsewhere changes in some domains of physical activity (occupational activity, transportation activity, domestic activity, and leisure sports activity) were associated with increased risk of obesity [17,38,39,40,45,46,47]. Decreased energy requirement resulted in drecreased total energy intake, majorly from decreased carbohydrate intake [20,21]. This study suggests that large increases in total fat intake, percentage of energy intake from fat, and percentage of high-fat diets may represent another reason.

There is no doubt that positive energy balance results in overweight and obesity. Excessive fat intake in China is an important contributor to positive energy balance, because increased dietary fat intake provides more energy from fat and total energy intake. A recent animal study showed that only increased dietary fat was associated with elevated total energy intake and body adiposity, while increased protein and carbohydrate intakes did not affect energy intake regulation or cause adiposity [48]. The mechanism hypothesized is that dietary fat increases the expression of the hypothalamic gene in reward pathways to increase energy intake and therefore body adiposity, which peaked in diets with 50–60% of energy from fat. However, results from this study need to be interpreted with caution, as C57BL/6 mice used in the models may be not sensitive to high-carbohydrate diet [49]. 

The consumption of a high-fat diet may decrease fat taste sensitivity and result in excessive fat intake. A recent six-week randomized dietary intervention study [50] showed that a low-fat diet reduced fat taste thresholds, or increased fat taste sensitivity, which likely helped induce a health satiety response to dietary fat and accordingly decreased body weight in people with overweight and obesity. A portion-control diet showed similar but weaker effects. A high-fat diet may induce hyperleptinemia and hyperinsulinemia accompanied by leptin and insulin resistance and lower suppression of ghrelin secretion [51]. 

The latest Dietary Guidelines for Americans removed the top limit for total fat intake [17]. However, our long-term China cohort study suggests that in China a high total fat intake significantly increases body weight and is associated with increased risk of overweight and obesity. Our findings are consistent with a six-month randomized controlled feeding trial that showed that a low-fat diet was less likely to increase body weight in nonobese adults than a high-fat diet [52]. It is important to note that high fat intake is not unique to China. Rather, fat intake has significantly increased in most Asian countries, including Indonesia and Malaysia [53]. 

While there is some consensus in the US. that many fatty foods are linked to increased risk of obesity and excessive weight gain, we cannot compare the effects of fat intake in the U.S. with those in China. First, our measurements of both diet and energy expenditure were taken with great detail. This includes measurement of all household recipes with precise disaggregation of each item in the dish, careful weighing of oil the household consumed, and detailed assessments of each domain of energy expenditure. For the dietary measures, we have shown that there is extreme variation in recipes within communities and provinces, and our approach allows us to collect individual recipes [23]. Such long-term data are not available in the U.S. Our data suggest that the diet shift in China is significantly different and that high edible oil intake is associated with increased adiposity. 

A study in Harbin City, China, indicated that long-term low-carbohydrate, high-fat, and high-protein diets were associated with increased risk of type 2 diabetes [54]. Energy intake from carbohydrate decreased significantly and caused the decrease in total energy intake in this study. However, to focus on the effects of fat intake, this study did not analyze the effects of energy intake from carbohydrates or from protein on the obesity epidemic in China. Instead, this study adjusted for total nonfat energy (i.e., energy from carbohydrate and energy from protein) in all fat intake models and adjusted for total energy intake in percentage of energy intake from fat and in high-fat diet models to minimize the bias. This study also tested if the magnitudes of the coefficients for total fat intake and percentage of energy intake from fat were significantly greater than the coefficients for total energy intake or for total nonfat energy intake.

The present study breaks new ground by exploring 25-year trends in high-fat diets and their associations with body weight and the risk of overweight and obesity among Chinese adults. The findings can inform policy makers in their development of programs to prevent obesity in Chinese adults. This is the only study to date that provides a longitudinal analysis of fat intake and overweight and obesity in China. No other study has had the size and heterogeneity to evaluate the impact of fat intake on body weight. Nonetheless, this study has some limitations. First, previous studies showed that saturated fat, rather than unsaturated fat or the amount of fat, increased the risk of cardiovascular disease events and related deaths [17]. We do not have the data on types of fatty acids and could not explore the relationships between the types of fatty acids and overweight and obesity. This study actually collected all edible oils found in the survey provinces, so many foods were fraudulently mislabeled regarding in their actual oils; we realized that without measurement of the oil in each household we could not use these individual oil data [55]. However, although quality of fat (e.g., saturated fatty acids) may be of some relevance, this conclusion is still hotly debated, as some metabolites of unsaturated fat may be harmful. Some recent studies revealed that oxidized derivatives of linoleic acid (18:2), a common component of vegetable oils, increase the risk of coronary heart disease and type 2 diabetes [56]. 

Second, this study may have underestimated fat intake in recent survey years. Away-from-home eating, snacking, and processed food consumption are increasing rapidly in China [5,57], and the Chinese FCT does not accurately capture the varying fat contents of these foods and dishes. This is a common problem globally, as FCTs are slow to reflect fat changes in packaged processed foods and food consumed away from home. This study minimized many common reporting errors by weighing all foods, including edible oils and seasonings, consumed over a three-day period, measuring the details of each recipe and cooking method, and decomposing the exact ingredients each household used in all prepared dishes each day. Third, lost-to-follow-up and replenishment participants may have biased the results. In other research our team has found that these issues did not significantly bias our analyses of the incidence of the many cardiometabolic problems we have studied [58]. Fourth, nonconsecutive 24 h dietary recalls may better estimate usual dietary intake distributions [59], but this is not practical in large multipurpose epidemiological surveys like ours. Numerous studies have shown that multiple recalls can significantly increase the accuracy of dietary intakes and decrease attenuation of the RR estimates [60,61].

## 5. Conclusions

In conclusion, high fat intake and high energy intake from fat are positively associated with body weight, BMI, and the risk of overweight and obesity in Chinese adults. Findings from studies in the U.S. that dietary fat intake has been constantly high and did not significantly change in the past five decades [42,62] may not apply to the Chinese population or other Asian populations where both fat intake and the prevalence of overweight and obesity are increasing. Western strategies to prevent and control overweight and obesity may be ineffective in China. Reducing dietary fat intake is a public health priority and an opportunity for China to improve its population’s health and quality of life. Public health approaches to prevent cardiovascular diseases may benefit from using these data to tailor programs accordingly. The findings of this study can have a significant impact on interventions to control overweight and obesity.

## Figures and Tables

**Table 1:** Sample characteristics of the China Health and Nutriton Survey, 1991–2015.

		1991	2000	2011	2015	*p* Trend
*n*	Total	6712	7258	8692	9338	
*n* (%)	Men	3139 (46.8)	3448 (47.5)	4011 (46.1)	4210 (45.1)	
*n* (%)	Women	3573 (53.2)	3810 (52.5)	4681 (53.9)	5128 (54.9)	
Age (years) ^1^	Average	37.6 (10.9)	40.5 (10.4)	44.2 (10.5)	44.4 (10.1)	<0.001
	Men	37.8 (10.9)	40.5 (10.6)	44.4 (10.5)	44.8 (10.0)	<0.001
	Women	37.5 (10.9)	40.5 (10.2)	44.0 (10.6)	44.1 (10.1)	<0.001
Urban residents (%)	Average	32.1	29.0	41.8	37.0	<0.001
	Men	31.5	28.5	41.5	36.7	<0.001
	Women	32.5	29.4	42.0	37.3	<0.001
Education ≥ high school (%)	Average	17.4	20.0	38.8	41.1	<0.001
	Men	21.0	24.4	41.9	45.0	<0.001
	Women	14.3	15.9	36.1	37.9	<0.001
Income (1000 yuan) ^1,2^	Average	3.3 (2.4)	6.2 (6.2)	17.3 (18.6)	23.7 (38.6)	<0.001
	Men	3.3 (2.5)	6.2 (6.2)	17.9 (19.7)	24.3 (39.7)	<0.001
	Women	3.3 (2.4)	6.2 (6.2)	16.8 (17.7)	23.2 (37.7)	<0.001
Smoker (%)	Average	35.5	32.1	30.0	26.3	<0.001
	Men	71.8	63.8	62.6	56.5	<0.001
	Women	3.5	3.5	2.1	1.4	<0.001
Drinker (%)	Average	38.8	35.7	36.6	29.9	<0.001
	Men	68.1	64.7	64.6	57.4	<0.001
	Women	13.0	9.4	12.7	7.3	<0.001
Physical activities (MET hrs./wk.) ^1,3^	Average	62.3 (37.6)	41.5 (30.7)	27.9 (26.8)	21.7 (24.1)	<0.001
	Men	57.2 (34.8)	39.7 (29.6)	27.9 (26.8)	22.3 (25.3)	<0.001
	Women	66.7 (39.3)	43.2 (31.5)	28.0 (26.9)	21.3 (23.0)	<0.001

^1^ Values are given as mean (standard deviation). ^2^ Chinese yuan adjusted to 2015 values. ^3^ Metabolic Equivalent of Task (MET) hours per week.

**Table 2:** Fat intake and prevalence of overweight and obesity, China Health and Nutrition Survey, 1991–2015 ^1^.

		1991	2000	2011	2015	*p* Trend
Energy intake (kcal/d)	Average	2712.2 (708.3)	2409.6 (684.1)	2005.9 (660.7)	2003.1 (671.3)	<0.001
	Men	2916.9 (722.1)	2605.2 (688.2)	2196.9 (686.2)	2186.4 (706.0)	<0.001
	Women	2532.4 (644.5)	2232.6 (630.1)	1842.3 (590.9)	1852.6 (601.0)	<0.001
Fat intake (g/d)	Average	67.4 (36.4)	78.2 (38.3)	77.5 (33.7)	78.3 (36.9)	<0.001
	Men	71.5 (37.9)	82.9 (39.5)	83.0 (35.2)	83.9 (37.8)	<0.001
	Women	63.9 (34.8)	73.9 (36.7)	72.7 (31.7)	73.7 (35.6)	<0.001
% fat intake from edible oil	Average	50.5 (22.9)	52.9 (23.0)	44.8 (21.2)	43.0 (22.9)	<0.001
	Men	48.9 (22.9)	51.8 (23.0)	43.5 (20.8)	41.7 (22.8)	<0.001
	Women	52.0 (22.7)	53.9 (23.0)	45.9 (21.4)	44.0 (22.8)	<0.001
% energy intake from fat	Average	22.4 (10.0)	28.9 (10.4)	35.1 (10.9)	35.3 (11.4)	<0.001
	Men	22.1 (9.9)	28.3 (10.3)	34.4 (10.8)	34.8 (11.2)	<0.001
	Women	22.7 (10.0)	29.4 (10.5)	35.7 (10.9)	35.7 (11.6)	<0.001
% energy intake from edible oil	Average	11.1 (7.0)	15.1 (8.6)	16.0 (9.9)	15.6 (10.7)	<0.001
	Men	10.6 (6.9)	14.5 (8.3)	15.2 (9.4)	14.9 (10.4)	<0.001
	Women	11.6 (7.2)	15.6 (8.7)	16.7 (10.1)	16.2 (10.9)	<0.001
High-fat diet (%) ^2,3^	Average	22.4	44.2	67.0	67.2	<0.001
	Men	20.8	42.3	64.3	65.8	<0.001
	Women	23.8	45.9	69.4	68.5	<0.001
Height (cm)	Average	160.0 (8.1)	161.3 (8.1)	162.6 (8.4)	162.4 (8.2)	<0.001
	Men	165.7 (6.3)	167.0 (6.3)	168.7 (6.6)	168.3 (6.6)	<0.001
	Women	154.9 (5.8)	156.1 (5.8)	157.5 (6.0)	157.5 (5.8)	<0.001
Weight (kg)	Average	55.8 (9.0)	59.7 (10.3)	63.3 (11.8)	63.7 (11.8)	<0.001
	Men	59.2 (8.6)	63.8 (10.3)	68.6 (11.6)	69.3 (11.8)	<0.001
	Women	52.8 (8.2)	56.1 (8.9)	58.8 (9.9)	59.1 (9.7)	<0.001
BMI (kg/m^2^) ^4^	Average	21.7 (2.8)	22.9 (3.1)	23.9 (3.6)	24.1 (3.7)	<0.001
	Men	21.5 (2.5)	22.8 (3.0)	24.0 (3.4)	24.4 (3.6)	<0.001
	Women	22.0 (2.9)	23.0 (3.2)	23.7 (3.7)	23.8 (3.7)	<0.001
Overweight (BMI ≥ 25.0–29.9) ^2^	Average	11.2	21.2	28.7	31.2	<0.001
	Men	9.0	20.3	31.2	35.8	<0.001
	Women	13.2	22.0	26.5	27.5	<0.001
Obesity (BMI ≥ 30.0) ^2^	Average	1.1	2.3	5.2	6.0	<0.001
	Men	0.6	2.0	4.9	6.1	<0.001
	Women	1.5	2.6	5.5	6.0	<0.001
Overweight and obesity ^2^	Average	12.3	23.5	33.9	37.3	<0.001
	Men	9.7	22.4	36.1	41.9	<0.001
	Women	14.7	24.5	32.0	33.5	<0.001

^1^ Values are given as mean (standard deviation). ^2^ Values are given as percentages. ^3^ High-fat diet is defined as percentage of energy intake from fat > 30%. ^4^ Body Mass Index (BMI).

**Table 3:** Associations of fat intake and percentage of energy intake from fat with body weight, BMI, and the risk of overweight and obesity, China Health and Nutrition Survey, 1991–2015 ^1^.

**Model 1: Fat Intake (10 g/d Increase) ^2^**						
	Weight (kg) ^3^			BMI (kg/m^2^)		
	Coefficient	95% CI ^4^	*p* value	Coefficient	95% CI	*p* value
Average	0.030	0.018–0.041	<0.0001	0.011	0.007–0.016	<0.0001
Men	0.023	0.006–0.041	0.0080	0.009	0.003–0.015	0.0050
Women	0.033	0.018–0.049	<0.0001	0.014	0.007–0.020	<0.0001
**Model 2: % energy intake from fat (10% per day increase)**						
		Weight (kg) ^3^		BMI (kg/m^2^)		
	Coefficient	95% CI	*p* value	Coefficient	95% CI	p value
Average	0.092	0.051–0.133	<0.0001	0.038	0.022–0.054	<0.0001
Men	0.092	0.027–0.157	0.0060	0.035	0.012–0.058	0.0030
Women	0.098	0.045–0.150	<0.0001	0.041	0.020–0.063	<0.0001
**Model 3: High-fat diet (energy intake from fat > 30%) ^5^**						
Overweight and obesity	RR ^6^	95% CI	*p* value			
Average	1.13	1.04–1.23	0.003			
Men	1.14	1.01–1.29	0.032			
Women	1.13	1.01–1.26	0.031			

^1^ Adjusted for age, sex, physical activity tertiles, income tertiles, residency, smoking status, drinking status, and survey year. ^2^ Additionally adjusted for total nonfat energy. ^3^ Additionally adjusted for height. ^4^ 95% confidence interval (CI). ^5^ Additionally adjusted for total fat intake. ^6^ Relative risk (RR). Model 1: Association of fat intake (10 g/d increase) with body weight and Body Mass Index (BMI). Random-effects linear regression models were used. Model 2: Association of percentage of energy intake from fat (10% per day increase) with body weight and BMI. Random-effects linear regression models were used. Model 3: Association between a high-fat diet (energy intake from fat > 30%) and the risk of overweight and obesity. Random-effects logistic regression models were used.

## Supplementary Materials

The following are available online at https://www.mdpi.com/2072-6643/12/11/3272/s1. Table S1: Associations of fat intake with body weight, China Health and Nutrition Survey, 1991–2015. Table S2: Associations of fat intake with body weight among men, China Health and Nutrition Survey, 1991–2015. Table S3: Associations of fat intake with body weight among women, China Health and Nutrition Survey, 1991–2015. Table S4: Associations of fat intake with BMI, China Health and Nutrition Survey, 1991–2015. Table S5: Associations of fat intake with BMI among men, China Health and Nutrition Survey, 1991–2015. Table S6: Associations of fat intake with BMI among women, China Health and Nutrition Survey, 1991–2015. Table S7: Associations of energy intake from fat with body weight, China Health and Nutrition Survey, 1991–2015. Table S8: Associations of energy intake from fat with body weight among men, China Health and Nutrition Survey, 1991–2015. Table S9: Associations of energy intake from fat with body weight among women, China Health and Nutrition Survey, 1991–2015. Table S10: Associations of energy intake from fat with BMI, China Health and Nutrition Survey, 1991–2015. Table S11: Associations of energy intake from fat with BMI among men, China Health and Nutrition Survey, 1991–2015. Table S12: Associations of energy intake from fat with BMI among women, China Health and Nutrition Survey, 1991–2015. Table S13: Associations of a high-fat diet (energy intake from fat > 30%) with the risk of overweight and obesity, China Health and Nutrition Survey, 1991–2015. Table S14: Associations of a high-fat diet (energy intake from fat > 30%) with the risk of overweight and obesity among men, China Health and Nutrition Survey, 1991–2015. Table S15: Associations of a high-fat diet (energy intake from fat > 30%) with the risk of overweight and obesity among women, China Health and Nutrition Survey, 1991–2015.
