# Supplementary material for: Elevated Fat Intake Increases Body Weight and the Risk of Overweight and Obesity among Chinese Adults: 1991–2015 Trends
Source: Nutrients. 2020 Oct 26;12(11):3272. doi: 10.3390/nu12113272 (PMC7694029; doi:10.3390/nu12113272)
Supplement: Supplementary file 1 [file nutrients-12-03272-s001.pdf]

Table S1. Associations of fat intake with body weight, China Health and Nutrition Survey, 1991–2015

Variables		Coef.	Std. Err.	z	P>|z|	[95% Conf. Interval]	
						Lower	Upper
Fat intake (10 g)		0.030	0.006	4.970	0.000	0.018	0.041
Energy intake from nonfat (10%)		-0.002	0.004	-0.460	0.648	-0.010	0.007
Height (cm)		0.609	0.008	78.280	0.000	0.593	0.624
Age (years)		0.099	0.005	18.100	0.000	0.089	0.110
Female		-1.929	0.164	-11.760	0.000	-2.250	-1.607
High School		0.011	0.091	0.120	0.907	-0.169	0.190
Physical activities							
	Mid	-0.056	0.053	-1.050	0.293	-0.159	0.048
	High	-0.159	0.061	-2.600	0.009	-0.279	-0.039
Income							
	Mid	0.109	0.050	2.180	0.029	0.011	0.207
	High	0.299	0.058	5.150	0.000	0.185	0.413
Urban		1.292	0.143	9.040	0.000	1.012	1.573
Smoker		-0.543	0.077	-7.010	0.000	-0.695	-0.391
Drinker		0.160	0.059	2.720	0.006	0.045	0.275
Survey year							
	1993	0.495	0.076	6.540	0.000	0.347	0.644
	1997	1.441	0.086	16.760	0.000	1.272	1.609
	2000	2.529	0.092	27.360	0.000	2.347	2.710
	2004	3.043	0.107	28.580	0.000	2.835	3.252
	2006	3.149	0.115	27.420	0.000	2.924	3.374
	2009	3.618	0.127	28.570	0.000	3.370	3.866
	2011	4.456	0.134	33.150	0.000	4.193	4.720
	2015	5.381	0.156	34.510	0.000	5.075	5.686
_cons		-44.454	1.342	-33.120	0.000	-47.085	-41.823

Table S2. Associations of fat intake with body weight among men, China Health and Nutrition Survey, 1991–2015

Variables		Coef.	Std. Err.	z	P>|z|	[95% Conf. Interval]	
						Lower	Upper
Fat intake (10 g)		0.023	0.009	2.640	0.008	0.006	0.041
Energy intake from nonfat (10%)		0.000	0.006	0.010	0.991	-0.012	0.012
Height (cm)		0.698	0.011	60.750	0.000	0.676	0.721
Age (years)		0.059	0.008	7.290	0.000	0.043	0.075
High School		0.437	0.132	3.310	0.001	0.178	0.695
Physical activities							
	Mid	0.040	0.081	0.490	0.624	-0.120	0.199
	High	-0.152	0.096	-1.590	0.112	-0.340	0.035
Income							
	Mid	0.133	0.078	1.710	0.088	-0.020	0.285
	High	0.382	0.090	4.230	0.000	0.205	0.559
Urban		2.015	0.213	9.440	0.000	1.596	2.433
Smoker		-0.566	0.088	-6.450	0.000	-0.738	-0.394
Drinker		0.293	0.077	3.810	0.000	0.142	0.444
Survey year							
	1993	0.697	0.118	5.910	0.000	0.466	0.928
	1997	1.724	0.132	13.060	0.000	1.465	1.983
	2000	3.031	0.142	21.410	0.000	2.753	3.308
	2004	3.846	0.162	23.720	0.000	3.528	4.163
	2006	4.127	0.174	23.660	0.000	3.785	4.469
	2009	4.763	0.191	24.960	0.000	4.389	5.137
	2011	5.836	0.203	28.770	0.000	5.438	6.233
	2015	7.044	0.235	29.960	0.000	6.583	7.505
_cons		-59.081	1.983	-29.790	0.000	-62.969	-55.194

Table S3. Associations of fat intake with body weight among women, China Health and Nutrition Survey, 1991–2015

Variables		Coef.	Std. Err.	z	P>|z|	[95% Conf. Interval]	
						Lower	Upper
Fat intake (10 g)		0.033	0.008	4.160	0.000	0.018	0.049
Energy intake from nonfat (10%)		-0.002	0.006	-0.360	0.720	-0.014	0.010
Height (cm)		0.518	0.010	49.890	0.000	0.498	0.538
Age (years)		0.135	0.007	18.540	0.000	0.120	0.149
High School		-0.462	0.126	-3.660	0.000	-0.709	-0.214
Physical activities							
	Mid	-0.167	0.068	-2.440	0.015	-0.301	-0.033
	High	-0.257	0.079	-3.270	0.001	-0.411	-0.103
Income							
	Mid	0.093	0.064	1.450	0.146	-0.032	0.219
	High	0.232	0.075	3.100	0.002	0.085	0.378
Urban		0.619	0.188	3.290	0.001	0.250	0.987
Smoker		-0.164	0.217	-0.760	0.450	-0.588	0.261
Drinker		-0.088	0.094	-0.930	0.351	-0.272	0.097
Survey year							
1993		0.323	0.097	3.320	0.001	0.133	0.514
1997		1.190	0.111	10.680	0.000	0.972	1.409
2000		2.078	0.120	17.320	0.000	1.843	2.313
2004		2.321	0.139	16.710	0.000	2.049	2.593
2006		2.278	0.150	15.190	0.000	1.984	2.572
2009		2.607	0.166	15.690	0.000	2.282	2.933
2011		3.258	0.176	18.490	0.000	2.913	3.604
2015		3.933	0.204	19.250	0.000	3.533	4.334
_cons		-32.528	1.675	-19.420	0.000	-35.812	-29.245

Table S4. Associations of fat intake with BMI, China Health and Nutrition Survey, 1991–2015

Variables	Coef.	Std. Err.	z	P>|z|	[95% Conf. Interval]	
					Lower	Upper
Fat intake (10 g)	0.011	0.002	4.970	0.000	0.007	0.016
Energy intake from nonfat (100 kcal)	-0.001	0.002	-0.540	0.591	-0.004	0.002
Age (years)	0.044	0.002	21.860	0.000	0.040	0.048
Female	-0.125	0.053	-2.350	0.019	-0.229	-0.021
High School	-0.055	0.035	-1.570	0.116	-0.123	0.013
Physical activities						
Mid	-0.028	0.020	-1.380	0.169	-0.067	0.012
High	-0.072	0.023	-3.090	0.002	-0.118	-0.026
Income						
Mid	0.043	0.019	2.250	0.025	0.005	0.080
High	0.106	0.022	4.760	0.000	0.062	0.149
Urban	0.381	0.054	7.110	0.000	0.276	0.485
Smoker	-0.193	0.030	-6.520	0.000	-0.250	-0.135
Drinker	0.048	0.022	2.130	0.033	0.004	0.092
Survey year						
1993	0.178	0.029	6.150	0.000	0.121	0.235
1997	0.522	0.033	15.990	0.000	0.458	0.586
2000	0.922	0.035	26.360	0.000	0.853	0.990
2004	1.090	0.040	27.190	0.000	1.012	1.169
2006	1.106	0.043	25.680	0.000	1.022	1.191
2009	1.256	0.047	26.560	0.000	1.163	1.349
2011	1.567	0.050	31.230	0.000	1.469	1.666
2015	1.897	0.058	32.610	0.000	1.783	2.011
_cons	20.111	0.091	221.110	0.000	19.932	20.289

Table S5. Associations of fat intake with BMI among men, China Health and Nutrition Survey, 1991–2015

Variables		Coef.	Std. Err.	z	P>|z|	[95% Conf. Interval] Lower	Upper
Fat intake (10 g)		0.009	0.003	2.810	0.005	0.003	0.015
Energy intake from nonfat (100 kcal)		0.000	0.002	0.010	0.995	-0.004	0.004
Age (years)		0.024	0.003	8.620	0.000	0.019	0.030
High School		0.134	0.047	2.880	0.004	0.043	0.225
Physical activities							
	Mid	0.004	0.029	0.130	0.896	-0.053	0.060
	High	-0.061	0.034	-1.790	0.074	-0.127	0.006
Income							
	Mid	0.048	0.028	1.730	0.084	-0.006	0.102
	High	0.133	0.032	4.150	0.000	0.070	0.195
Urban		0.661	0.074	8.890	0.000	0.515	0.806
Smoker		-0.203	0.031	-6.500	0.000	-0.264	-0.142
Drinker		0.098	0.027	3.600	0.000	0.045	0.152
Survey year							
	1993	0.245	0.042	5.850	0.000	0.163	0.327
	1997	0.603	0.047	12.910	0.000	0.511	0.694
	2000	1.064	0.050	21.330	0.000	0.966	1.161
	2004	1.340	0.057	23.580	0.000	1.228	1.451
	2006	1.427	0.061	23.420	0.000	1.307	1.546
	2009	1.638	0.066	24.700	0.000	1.508	1.768
	2011	2.014	0.070	28.570	0.000	1.876	2.152
	2015	2.443	0.082	29.910	0.000	2.283	2.603
_cons		20.505	0.119	172.030	0.000	20.271	20.738

Table S6. Associations of fat intake with BMI among women, China Health and Nutrition Survey, 1991–2015

Variables		Coef.	Std. Err.	z	P>|z|	[95% Conf. Interval]	
						Lower	Upper
Fat intake (10 g)		0.014	0.003	4.080	0.000	0.007	0.020
Energy intake from nonfat (100 kcal)		-0.002	0.002	-0.770	0.441	-0.007	0.003
Age (years)		0.063	0.003	21.640	0.000	0.057	0.068
High School		-0.253	0.052	-4.890	0.000	-0.354	-0.151
Physical activities							
	Mid	-0.055	0.028	-1.950	0.052	-0.110	0.000
	High	-0.088	0.032	-2.720	0.007	-0.151	-0.024
Income							
	Mid	0.040	0.026	1.520	0.128	-0.012	0.092
	High	0.084	0.031	2.740	0.006	0.024	0.144
Urban		0.136	0.076	1.800	0.072	-0.012	0.284
Smoker		-0.110	0.089	-1.240	0.217	-0.284	0.064
Drinker		-0.035	0.039	-0.920	0.359	-0.111	0.040
Survey year							
	1993	0.118	0.040	2.960	0.003	0.040	0.197
	1997	0.445	0.046	9.750	0.000	0.356	0.535
	2000	0.788	0.049	16.120	0.000	0.693	0.884
	2004	0.860	0.056	15.270	0.000	0.749	0.970
	2006	0.812	0.061	13.420	0.000	0.694	0.931
	2009	0.909	0.067	13.610	0.000	0.778	1.040
	2011	1.167	0.071	16.490	0.000	1.028	1.306
	2015	1.406	0.082	17.150	0.000	1.245	1.567
_cons		19.623	0.118	165.790	0.000	19.391	19.855

Table S7. Associations of energy intake from fat with body weight, China Health and Nutrition Survey, 1991–2015

Variables		Coef.	Std. Err.	z	P>|z|	[95% Conf. Interval]	
						Lower	Upper
Energy intake from fat (10%)		0.092	0.021	4.350	0.000	0.051	0.133
Total energy intake (100 kcal)		0.009	0.003	2.680	0.007	0.002	0.015
Height (cm)		0.609	0.008	78.270	0.000	0.593	0.624
Age (years)		0.099	0.005	18.110	0.000	0.089	0.110
Female		-1.929	0.164	-11.760	0.000	-2.251	-1.608
High School		0.009	0.091	0.100	0.918	-0.170	0.189
Physical activities							
	Mid	-0.056	0.053	-1.060	0.290	-0.159	0.048
	High	-0.159	0.061	-2.600	0.009	-0.279	-0.039
Income							
	Mid	0.109	0.050	2.180	0.030	0.011	0.207
	High	0.300	0.058	5.170	0.000	0.186	0.414
Urban		1.291	0.143	9.030	0.000	1.011	1.571
Smoker		-0.544	0.077	-7.020	0.000	-0.696	-0.392
Drinker		0.159	0.059	2.710	0.007	0.044	0.275
Survey year							
	1993	0.498	0.076	6.580	0.000	0.350	0.646
	1997	1.445	0.086	16.860	0.000	1.277	1.613
	2000	2.536	0.092	27.620	0.000	2.356	2.716
	2004	3.052	0.106	28.800	0.000	2.844	3.259
	2006	3.154	0.114	27.570	0.000	2.929	3.378
	2009	3.621	0.126	28.700	0.000	3.374	3.868
	2011	4.451	0.134	33.150	0.000	4.188	4.714
	2015	5.374	0.156	34.490	0.000	5.069	5.680
_cons		-44.731	1.342	-33.330	0.000	-47.361	-42.101

Table S8. Associations of energy intake from fat with body weight among men, China Health and Nutrition Survey, 1991–2015

Variables	Coef.	Std. Err.	z	P>|z|	[95% Conf. Interval]	
					Lower	Upper
Energy intake from fat (10%)	0.092	0.033	2.770	0.006	0.027	0.157
Total energy intake (100 kcal)	0.008	0.005	1.610	0.108	-0.002	0.017
Height (cm)	0.698	0.011	60.750	0.000	0.676	0.721
Age (years)	0.059	0.008	7.290	0.000	0.043	0.075
High School	0.435	0.132	3.290	0.001	0.176	0.693
Physical activities						
Mid	0.041	0.081	0.500	0.616	-0.119	0.200
High	-0.151	0.096	-1.570	0.116	-0.338	0.037
Income						
Mid	0.130	0.078	1.670	0.094	-0.022	0.282
High	0.380	0.090	4.210	0.000	0.203	0.557
Urban	2.006	0.213	9.400	0.000	1.588	2.424
Smoker	-0.567	0.088	-6.450	0.000	-0.739	-0.395
Drinker	0.293	0.077	3.800	0.000	0.142	0.444
Survey year						
1993	0.696	0.118	5.900	0.000	0.465	0.927
1997	1.719	0.132	13.050	0.000	1.460	1.977
2000	3.024	0.141	21.500	0.000	2.748	3.299
2004	3.839	0.161	23.820	0.000	3.523	4.155
2006	4.115	0.174	23.700	0.000	3.775	4.455
2009	4.747	0.190	25.000	0.000	4.375	5.119
2011	5.810	0.202	28.710	0.000	5.413	6.206
2015	7.014	0.235	29.870	0.000	6.554	7.474
_cons	-59.318	1.983	-29.920	0.000	-63.204	-55.432

Table S9. Associations of energy intake from fat with body weight among women, China Health and Nutrition Survey, 1991–2015

Variables		Coef.	Std. Err.	z	P>|z|	[95% Conf. Interval]	
						Lower	Upper
Energy intake from fat (10%)		0.098	0.027	3.620	0.000	0.045	0.150
Total energy intake (100 kcal)		0.010	0.004	2.240	0.025	0.001	0.019
Height (cm)		0.518	0.010	49.870	0.000	0.497	0.538
Age (years)		0.135	0.007	18.550	0.000	0.121	0.149
High School		-0.463	0.126	-3.670	0.000	-0.711	-0.216
Physical activities							
	Mid	-0.167	0.068	-2.430	0.015	-0.301	-0.032
	High	-0.257	0.079	-3.270	0.001	-0.411	-0.103
Income							
	Mid	0.094	0.064	1.460	0.145	-0.032	0.219
	High	0.233	0.075	3.120	0.002	0.086	0.379
Urban		0.617	0.188	3.280	0.001	0.249	0.986
Smoker		-0.161	0.217	-0.740	0.457	-0.586	0.264
Drinker		-0.088	0.094	-0.940	0.347	-0.273	0.096
Survey year							
	1993	0.325	0.097	3.340	0.001	0.134	0.515
	1997	1.196	0.111	10.750	0.000	0.978	1.414
	2000	2.085	0.119	17.490	0.000	1.852	2.319
	2004	2.329	0.138	16.850	0.000	2.058	2.600
	2006	2.282	0.149	15.270	0.000	1.989	2.575
	2009	2.609	0.166	15.750	0.000	2.285	2.934
	2011	3.251	0.176	18.460	0.000	2.906	3.597
	2015	3.926	0.204	19.220	0.000	3.525	4.326
_cons		-32.815	1.675	-19.590	0.000	-36.098	-29.532

Table S10. Associations of energy intake from fat with BMI, China Health and Nutrition Survey, 1991–2015

Variables		Coef.	Std. Err.	z	P>|z|	[95% Conf. Interval]	
						Lower	Upper
Energy intake from fat(10%)		0.038	0.008	4.680	0.000	0.022	0.054
Total energy intake (100 kcal)		0.003	0.001	2.600	0.009	0.001	0.006
Age (years)		0.044	0.002	21.870	0.000	0.040	0.048
Female		-0.125	0.053	-2.360	0.018	-0.229	-0.021
High School		-0.055	0.035	-1.590	0.112	-0.123	0.013
Physical activities							
	Mid	-0.028	0.020	-1.370	0.170	-0.067	0.012
	High	-0.072	0.023	-3.080	0.002	-0.118	-0.026
Income							
	Mid	0.042	0.019	2.230	0.026	0.005	0.080
	High	0.106	0.022	4.770	0.000	0.062	0.149
Urban		0.379	0.054	7.080	0.000	0.274	0.484
Smoker		-0.193	0.030	-6.530	0.000	-0.251	-0.135
Drinker		0.047	0.022	2.110	0.035	0.003	0.091
Survey year							
	1993	0.178	0.029	6.180	0.000	0.122	0.235
	1997	0.523	0.033	16.050	0.000	0.459	0.587
	2000	0.923	0.035	26.560	0.000	0.855	0.991
	2004	1.092	0.040	27.370	0.000	1.014	1.170
	2006	1.106	0.043	25.790	0.000	1.022	1.190
	2009	1.255	0.047	26.630	0.000	1.162	1.347
	2011	1.562	0.050	31.170	0.000	1.464	1.661
	2015	1.891	0.058	32.540	0.000	1.777	2.005
_cons		19.996	0.091	220.340	0.000	19.818	20.174

Table S11. Associations of energy intake from fat with BMI among men, China Health and Nutrition Survey, 1991–2015

Variables		Coef.	Std. Err.	z	P>|z|	[95% Conf. Interval]	
						Lower	Upper
Energy intake from fat(10%)		0.035	0.012	2.960	0.003	0.012	0.058
Total energy intake (100 kcal)		0.003	0.002	1.700	0.089	0.000	0.006
Age (years)		0.024	0.003	8.610	0.000	0.019	0.030
High School		0.133	0.047	2.860	0.004	0.042	0.225
Physical activities							
	Mid	0.004	0.029	0.140	0.886	-0.052	0.061
	High	-0.060	0.034	-1.770	0.077	-0.127	0.006
Income							
	Mid	0.047	0.028	1.690	0.091	-0.007	0.101
	High	0.132	0.032	4.120	0.000	0.069	0.195
Urban		0.657	0.074	8.840	0.000	0.511	0.803
Smoker		-0.203	0.031	-6.510	0.000	-0.264	-0.142
Drinker		0.098	0.027	3.590	0.000	0.045	0.152
Survey year							
	1993	0.244	0.042	5.840	0.000	0.162	0.326
	1997	0.601	0.047	12.900	0.000	0.510	0.692
	2000	1.061	0.050	21.420	0.000	0.964	1.158
	2004	1.337	0.056	23.670	0.000	1.226	1.448
	2006	1.422	0.061	23.460	0.000	1.303	1.541
	2009	1.632	0.066	24.720	0.000	1.503	1.762
	2011	2.004	0.070	28.480	0.000	1.866	2.142
	2015	2.431	0.082	29.800	0.000	2.271	2.591
_cons		20.410	0.119	171.020	0.000	20.176	20.643

Table S12. Associations of energy intake from fat with BMI among women, China Health and Nutrition Survey, 1991–2015

Variables		Coef.	Std. Err.	z	P>|z|	[95% Conf. Interval]	
						Lower	Upper
Energy intake from fat (10%)		0.041	0.011	3.720	0.000	0.020	0.063
Total energy intake (100 kcal)		0.003	0.002	1.850	0.065	0.000	0.007
Age (years)		0.063	0.003	21.650	0.000	0.057	0.068
High School		-0.253	0.052	-4.910	0.000	-0.354	-0.152
Physical activities							
	Mid	-0.055	0.028	-1.950	0.051	-0.110	0.000
	High	-0.088	0.032	-2.730	0.006	-0.151	-0.025
Income							
	Mid	0.040	0.026	1.530	0.127	-0.011	0.092
	High	0.085	0.031	2.760	0.006	0.025	0.145
Urban		0.136	0.076	1.790	0.073	-0.013	0.284
Smoker		-0.109	0.089	-1.220	0.221	-0.283	0.066
Drinker		-0.036	0.039	-0.920	0.355	-0.112	0.040
Survey year							
	1993	0.119	0.040	2.980	0.003	0.041	0.197
	1997	0.448	0.046	9.830	0.000	0.359	0.537
	2000	0.792	0.049	16.300	0.000	0.697	0.888
	2004	0.864	0.056	15.430	0.000	0.754	0.974
	2006	0.815	0.060	13.510	0.000	0.697	0.933
	2009	0.911	0.067	13.680	0.000	0.780	1.041
	2011	1.165	0.071	16.470	0.000	1.026	1.304
	2015	1.404	0.082	17.140	0.000	1.244	1.565
_cons		19.490	0.118	164.920	0.000	19.259	19.722

Table S13. Associations of a high-fat diet (energy intake from fat > 30%) with the risk of overweight and obesity, China Health and Nutrition Survey, 1991–2015

Variables		Odds Ratio	Std. Err.	z	P>|z|	[95% Conf. Interval]	
						Lower	Upper
High-fat diet		1.132	0.047	2.980	0.003	1.043	1.228
Total energy intake (100 kcal)		1.004	0.003	1.380	0.167	0.998	1.010
Age (years)		1.064	0.004	17.860	0.000	1.057	1.071
Female		0.818	0.075	-2.180	0.029	0.683	0.980
High School		0.812	0.060	-2.840	0.004	0.703	0.937
Physical activities							
	Mid	0.871	0.042	-2.840	0.004	0.792	0.958
	High	0.791	0.044	-4.190	0.000	0.709	0.883
Income							
	Mid	1.124	0.054	2.430	0.015	1.023	1.236
	High	1.280	0.069	4.560	0.000	1.151	1.423
Urban		1.770	0.158	6.380	0.000	1.485	2.109
Smoker		0.722	0.049	-4.780	0.000	0.631	0.825
Drinker		1.068	0.059	1.190	0.233	0.959	1.189
Survey year							
	1993	1.378	0.121	3.650	0.000	1.160	1.637
	1997	2.873	0.264	11.490	0.000	2.400	3.440
	2000	5.608	0.514	18.830	0.000	4.687	6.711
	2004	7.642	0.745	20.870	0.000	6.313	9.251
	2006	7.354	0.739	19.860	0.000	6.039	8.954
	2009	9.678	1.016	21.610	0.000	7.878	11.890
	2011	14.614	1.583	24.770	0.000	11.819	18.070
	2015	23.516	2.813	26.400	0.000	18.602	29.729
_cons		0.001	0.000	-38.340	0.000	0.000	0.001

Table S14. Associations of a high-fat diet (energy intake from fat > 30%) with the risk of overweight and obesity, China Health and Nutrition Survey, 1991–2015

Variables	Odds Ratio	Std. Err.	z	P>|z|	[95% Conf. Interval] Lower	Upper
High-fat diet	1.140	0.070	2.140	0.032	1.011	1.285
Total energy intake (100 kcal)	1.002	0.004	0.570	0.567	0.994	1.011
Age (years)	1.033	0.005	6.490	0.000	1.023	1.043
High School	1.125	0.113	1.170	0.242	0.924	1.369
Physical activities						
Mid	0.808	0.057	-3.010	0.003	0.703	0.928
High	0.657	0.056	-4.940	0.000	0.556	0.776
Income						
Mid	1.154	0.084	1.970	0.049	1.001	1.331
High	1.400	0.113	4.150	0.000	1.194	1.641
Urban	2.791	0.362	7.900	0.000	2.164	3.600
Smoker	0.719	0.053	-4.470	0.000	0.622	0.831
Drinker	1.183	0.082	2.440	0.015	1.033	1.354
Survey year						
1993	1.562	0.220	3.160	0.002	1.184	2.059
1997	3.812	0.558	9.140	0.000	2.862	5.079
2000	9.487	1.380	15.460	0.000	7.133	12.618
2004	15.082	2.328	17.580	0.000	11.145	20.410
2006	16.620	2.631	17.750	0.000	12.186	22.666
2009	25.197	4.133	19.670	0.000	18.270	34.750
2011	41.598	7.078	21.910	0.000	29.802	58.063
2015	84.250	15.761	23.700	0.000	58.389	121.565
_cons	0.001	0.000	-26.020	0.000	0.000	0.001

Table S15. Associations of a high-fat diet (energy intake from fat > 30%) with the risk of overweight and obesity among women, China Health and Nutrition Survey, 1991–2015

Variables		Odds Ratio	Std. Err.	z	P>|z|	[95% Conf. Interval] Lower	Upper
High-fat diet		1.130	0.064	2.160	0.031	1.012	1.262
Total energy intake (100 kcal)		1.006	0.004	1.320	0.188	0.997	1.015
Age (years)		1.092	0.005	18.090	0.000	1.082	1.103
High School		0.548	0.059	-5.550	0.000	0.443	0.678
Physical activities							
	Mid	0.916	0.061	-1.320	0.187	0.804	1.044
	High	0.869	0.065	-1.870	0.061	0.750	1.007
Income							
	Mid	1.107	0.071	1.580	0.115	0.976	1.255
	High	1.194	0.087	2.440	0.015	1.035	1.377
Urban		1.185	0.146	1.380	0.168	0.931	1.509
Smoker		0.932	0.184	-0.360	0.722	0.633	1.373
Drinker		0.920	0.086	-0.890	0.375	0.766	1.106
Survey year							
	1993	1.265	0.142	2.090	0.037	1.015	1.577
	1997	2.329	0.277	7.110	0.000	1.845	2.940
	2000	3.774	0.450	11.150	0.000	2.988	4.767
	2004	4.524	0.577	11.830	0.000	3.523	5.810
	2006	3.864	0.511	10.220	0.000	2.982	5.008
	2009	4.496	0.628	10.760	0.000	3.419	5.911
	2011	6.326	0.907	12.870	0.000	4.776	8.378
	2015	8.292	1.316	13.330	0.000	6.075	11.318
_cons		0.000	0.000	-32.250	0.000	0.000	0.000
